# Characterization of novel and large fragment deletions in exon 1 of the *IL10RA* gene in Chinese children with very early onset inflammatory bowel diseases

**DOI:** 10.1186/s12876-021-01756-y

**Published:** 2021-04-13

**Authors:** Zifei Tang, Ping Zhang, Min Ji, Chunlan Yin, Ruiqin Zhao, Zhiheng Huang, Ying Huang

**Affiliations:** 1 Department of Gastroenterology, Children’s Hospital of Fudan University, 399 Wanyuan Road, Minhang District, Shanghai, 201102 China; 2 Center for Molecular Medicine, Pediatrics Research Institute, Children’s Hospital of Fudan University, Shanghai, 201102 China; 3 Department of Radiology, Children’s Hospital of Fudan University, Shanghai, 201102 China; 4 Department of Gastroenterology, Children’s Hospital of Hebei Province, Shijiazhuang, 050030 China

**Keywords:** Very early onset inflammatory bowel disease, Interleukin 10 receptor A, Large fragment deletions, Exon 1

## Abstract

**Background:**

Defects in interleukin 10 (IL10) and its receptors are particularly involved in very early onset inflammatory bowel disease (VEOIBD). However, large fragment deletions of IL10 receptor A (*IL10RA*) are rare.

**Methods:**

VEOIBD patients with confirmed mutations in the *IL10RA* gene were enrolled from January 1, 2019 to June 30, 2020. The clinical features and endoscopic-radiological findings of the patients with large fragment deletions of the *IL10RA* gene were determined and followed up.

**Results:**

Thirty-five patients with *IL10RA* gene mutations, namely, 28 compound heterozygous mutations and 7 homozygote mutations, were enrolled in this study. Six patients carried the reported point mutation c.301C > T (p. R101RW) or c.537 G > A (p. T179T) in one locus and a large fragment deletion in exon 1 in another locus, which were novel mutations in this gene. A 333-bp deletion of exon 1 (117857034–11857366 del) was the main mutation in this locus in 85.7% of the patients with large fragment deletions. The time of disease onset ranged from birth to 4 years, and diarrhea was the main initial symptom. In total, 6/7 patients had perianal complications, including perianal abscess, fistula and skin tags. Six patients accepted thalidomide treatment, 5/7 accepted mesalamine, 3/7 accepted hematopoietic stem cell transplantation (HSCT), and 3/7 were waiting for HSCT.

**Conclusions:**

We identified a novel large deletion of exon 1 involving the *IL10RA* gene for the first time and showed the characteristics of VEOIBD patients. This study expands the spectrum of Chinese VEOIBD patients with *IL0RA* gene mutations.

## Background

Inflammatory bowel disease (IBD) is a chronic relapsing disorder of the gastrointestinal tract with multifactorial and complex etiology. Very early onset IBD (VEOIBD) has an age of onset before 6 years old and constitutes 3–15% of pediatric IBD. VEOIBD is often associated with monogenetic disorders and is of particular interest in IBD research. Deficiencies in interleukin 10 (*IL10*) and its receptors (*IL10RA, IL0RB*) are major causes of VEOIBD [1, 2]. IL-10-related genes are most frequently associated with infantile-onset IBD (age of onset ≤ 2 years).

VEOIBD patients with mutations in the IL10 or IL10R genes present with severe gastrointestinal symptoms, such as severe colitis with hematochezia, severe perianal abscess or fistulae, repeated oral ulcer, and recurrent clinical and folliculitis in the first months of life [3]. These patients are refractory to immunosuppressive therapies such as corticosteroids, methotrexate, and antitumor necrosis factor-alpha (TNF-α) antibodies [4, 5]. For patients with severe intestinal infection or perianal lesions, enterostomy can alleviate the symptoms. Because IL10R is expressed on most hematopoietic cells, hematopoietic stem cell transplantation (HSCT) is considered the only curative option for IL10RA-deficient VEOIBD.

Many point mutations of *IL10* and *IL10R* have been identified at our IBD center and by other groups [6–8]. A previous study showed that *IL10RA* defects were unique genetic mutations in East Asia compared with North America and Europe, with the common point mutations being c.301C > T and c.537 G > A [9, 10]. The determination of the pathogenicity of candidate variants of the IL10RA gene is critical for the diagnosis and management of this disease. However, reports of large deletions in the *IL10RA* genes and their associated clinical characteristics are rare, and consequently some patients may never receive an appropriate treatment. In this study, we aimed to determine the characteristics of Chinese VEOIBD patients with large fragment deletions of the *IL10RA* gene.

## Methods

### Patient cohort

This study was approved by the Ethics Committee of the Children’s Hospital of Fudan University. Inpatients were recruited from a tertiary care center following a diagnosis of IBD based on their clinical history, physical examination, endoscopic appearance, and histological findings according to the Porto criteria. The index dates of the IBD diagnosis were from January 1, 2019 to June 30, 2020. Fifteen VEOIBD patients with IL10RA gene point mutations in the same period were enrolled as the control group. Patients with an infection or celiac disease or allergic/eosinophilic gastrointestinal diseases were excluded.

### Genetic analysis

Whole-exome sequencing (WES) was performed as described previously [11]. Target gene capture and next-generation sequencing (NGS) were also used to analyze the patients. The gene panel (MyGenostics, Beijing, China) contained 347 genes related to diarrhea. The library was prepared using the Library Preparation Kit developed by MyGenostics Co., Ltd. according to the Illumina platform requirements. High-throughput sequencing was performed using an Illumina × 10 sequencer. Exon 1 deletion was found by CapCNV analysis, followed by a CNVkit protocol (https://cnvkit.readthedocs.io/en/stable/pipeline.html).

### PCR and sanger sequencing

To verify the mutation sites detected by WES or NGS, the following procedure was used. The 200-bp DNA fragment including this site was amplified by polymerase chain reaction (PCR) and sequenced using an ABI 3730 Genetic Analyzer (Applied Biosystems, CA). To verify the exon 1 deletion, the DNA fragment including exon 1 was amplified by PCR using special primers, and two bands were obtained by agarose gel electrophoresis for the patients and their parents. The mutated DNA sequences were analyzed by BLAST in UCSC to confirm the deleted region. When identifying the novel sequence variant, pathogenicity was determined based on the American College of Medical Genetics and Genomics (ACMG) Standards and Guidelines [12].

### Laboratory indices

The erythrocyte sedimentation rate, C-reactive protein level, complete blood cell count, hemoglobin, total protein and albumin levels, and prealbumin level were recorded at the time of diagnosis. The available height for age (HFA), weight for age (WFA), and body mass index (BMI) Z score at the time of the first diagnosis for each patient were calculated using the World Health Organization (WHO) Anthro (version 3.2.2) software. All the patients with large fragment deletions of the *IL10RA* gene in this study had undergone follow-up for treatment and prognostic information.

The data were analyzed using SPSS 24.0 for Windows (SPSS Inc., Chicago, IL, USA). Continuous data are presented as the mean and standard deviation (SD) or the median and interquartile range (IQR). Categorical variables are reported as frequencies and percentages. A two-tailed value for *P* < 0.05 was considered statistically significant.

## Results

### Demographics of the patients

**Fig. 1:**
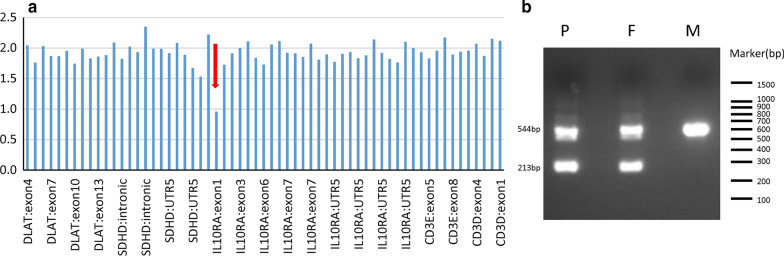
**a** Exon 1 deletion detected by NGS CapCNV analysis of patient 1. **b** PCR products of the exon 1 region of the IL10RA gene from patient 1 and his parents (for the original, full-length gel and lot images, see additional files). P = patient, F = father, M = mother

From January 1, 2019 to June 30, 2020, 35 patients with *IL10RA* gene mutations, including 33 compound heterozygous mutations and 2 homozygote mutations, were enrolled in the study. Seven patients with compound heterozygous mutations were found to carry point mutations c.301C > T (p. R101RW), c.537 G > A (p. T179T) or c.106G > A (p. A36T) in one locus and a large fragment deletion of the *IL10RA* gene in another locus (Table 1). The deletions in these 7 patients were all located in exon 1 of the *IL10RA* gene. Six patients (cases 1, 3, 4, 5, 6 and 7) (85.7%) had the same 333-bp deletion. The pathogenic evidence of this 333-bp deletion and untested mutation was PVS1 + PM2. To determine the impact of the 333-bp deletion on mRNA production, we performed PCR analysis in the index patient as well as his parents. Agarose gel electrophoresis showed an abnormal band in the proband and his father, and a normal band was seen in his mother. A representative figure of the exon 1 deletion of patient 1 is shown in Fig. 1a, b.

**Table 1:** The baseline information of the VEOIBD patients with *IL10RA* gene mutations

No	Sex	Consanguinity	Onset age, initial symptom	Main symptom	Perianal complications	Paris classification
1	M	NO	4 m, diarrhea	Diarrhea, bloody stool	Skin tag	A1aL3B1G1
2	F	NO	10 d, oral ulcer, eczema	Diarrhea	Abscess	A1aL2B1G0
3	M	NO	18 d, fever	Diarrhea, eczema	Abscess	A1aL3B1G1
4	M	NO	4 y, bloody stool	Diarrhea, bloody stool	–	A1aL3B1G0
5	M	NO	2 m, diarrhea	Diarrhea	Abscess, fistula	A1aL2B1G1
6	M	NO	14d, fever	Diarrhea, oral ulcer, eczema	Abscess, fistula	A1aL2B1G0
7	F	NO	0 d, fever	Diarrhea, eczema	Abscess, fistula	A1aL2B2G1

*BMI* body mass index, *d* day, *F* female, *HFA* height for age, *M* male, *m* month, *WFA* weight for age, *y* year

More detailed clinical characteristics of the 7 patients with identified mutations in *IL10RA* are shown in Table 2. For patient 4, although the disease onset was at 4 years of age, he still had refractory diarrhea and a bloody stool.

**Table 2:** The *IL10RA* gene information of the VEOIBD patients

No	*IL10RA*	Father	Mother	Siblings
1	Exon 1: chr11:117857034–117857366 del Exon 3: c.301C > T (p.R101W)	Exon 1:117857034–11857366 del	Exon 3: c.301C > T (p.R101W)	No
2	Exon 4: c.537G > A (p.179 T) Exon 1: chr11:117857182–117857249 del	Exon 4: c.537G > A (p.179 T)	Exon1:117857182–117857249 del	Old sister, health, undetected
3	Exon 1: chr11:117857034–117857366 del Exon 4: c.537G > A (p.179 T)	Exon 1:117857034–117857366 del	Exon4: c.537G > A (p.179 T)	No
4	Exon 4:c.537G > A (p.T179T) Exon 1: chr11:117857034–117857366 del	Exon 4:c.537G > A (p.T179T)	Exon 1:117857034–117857366 del	No
5	Exon 1: chr11:117857034–117857366 del Exon 3: c.301C > T (p.R101W)	Exon 1:117857034–11857362 del	Exon 3: c.301C > T (p.R101W)	Old sister, health, carrier Exon 3: c.301C > T (p.R101W)
6	Exon 1: chr11:117,857,034–117,857,366 del Exon 3: c.301C > T (p.R101W)	Exon 1:117857034–11857362 del	Exon 3: c.301C > T (p.R101W)	no
7	Exon 1: chr11:117857034–117857366 del Exon 2: c.106G > A(p.A36T)	Exon 2:c.106G > A(p.A36T)	Exon 1: chr11:117857034–117857366 del	Old sister, health, undetected

We compared the characteristic features of patients with a large fragment deletion in exon 1 of the IL10RA gene with 15 point mutations of the IL10RA gene. Both the patients with a large fragment deletion and the control patients had complete clinical data and were diagnosed with Crohn’s disease by endoscopy.

The laboratory data of these patients at the first outpatient visit to the hospital were collected. Although no significant difference was found, both groups with *IL10RA* gene mutations displayed increased white blood cell counts, C-reactive protein levels, platelet counts, erythrocyte sedimentation rates, and fecal calprotectin levels, while the expression levels of Hb, serum total protein, albumin and prealbumin were decreased. The patients with *IL10RA* mutations showed decreases in the average HFA, WFA and BMI (Table 3). The patients with large deletions in exon 1 were compared with other patients with the IL10RA gene point mutation. We could not find a significant difference between the two groups except on the day of diarrhea and the percentage of eczema.

**Table 3:** The comparison between large fragment deletions group and control group

Patients	Large fragment deletions group	Point mutation group	*P*
Body weight (kg)	8.1 ± 5.6	5.6 ± 1.6	0.12
Age of disease onset (day)	60.0[7.0,180.0]	14.0[9.0,20.0]	0.27
Age of diagnosis (day)	510.0[97.0,1099.0]	217.0[71.0,259.0]	0.09
*Symptom*			
Diarrhea (day)	60.0[16.0,180.0]	10.0[1.0,22.0]	0.01
Perianal lesions: n (%)	6(85.7)	12(80)	0.26
Oral ulcer: n (%)	1(14.3)	6(40)	0.23
Eczema: n (%)	6(85.7)	5(33.3)	0.02
HFA Z-score	− 2.9 ± 2.9	− 2.0 ± 1.1	0.45
WFA Z-score	− 2.6 ± 2.1	− 2.3 ± 1.2	0.66
*BMI Z-score*			
Simplified endoscopic score (SES)	22.0 ± 5.5	19.9 ± 8.5	0.57
*Laboratory tests*			
WBC	14.0 ± 4.8	15.1 ± 6.2	0.66
Hb	100.1 ± 19.4	97.9 ± 18.9	0.80
Platelet	518.3 ± 174.5	417.5 ± 152.9	0.18
CRP	19.0[9.0,55.4]	26.5[12.0,71.8]	0.78
ESR	34.0[13.0,50.0]	30.0[18.5,42.5]	0.94
Albumin	30.4 ± 4.1	30.8 ± 5.4	0.89
Prealbumin	105.3 ± 51.8	99.8 ± 61.5	0.84

### Endoscopic and imaging findings

**Fig. 2:**
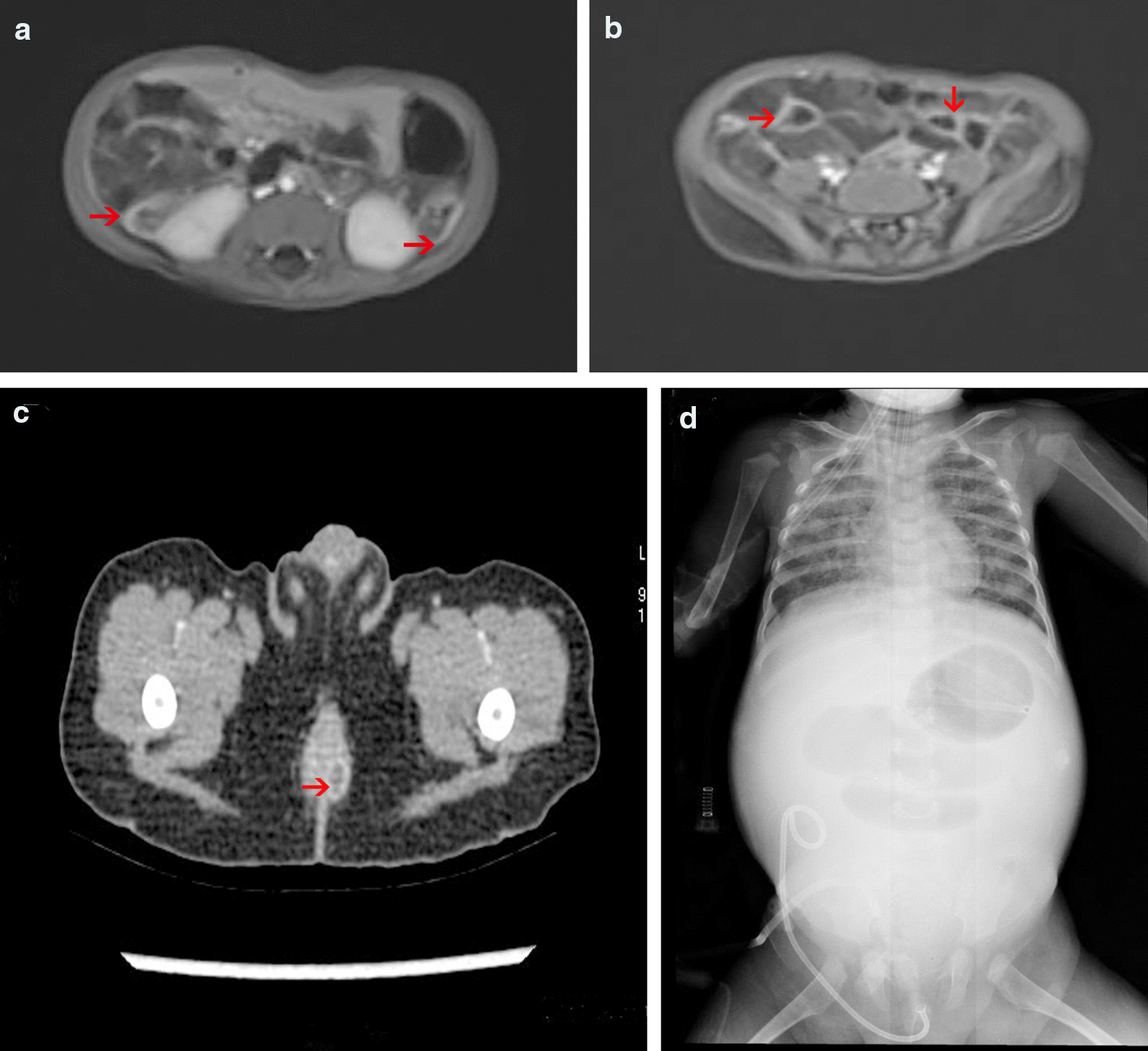
Imaging examination of VEOIBD patients with *IL10RA* gene mutations. **a** High signal of the colon in the abdominal MRI image of case 1. **b** High signal of the small bowel in the abdominal MRI image of case 1. **c** Perianal abscess in the enhanced CT image of case 3. **d** Intestinal obstruction and necrosis on X-ray examination in case 3

Endoscopy revealed that patient 1 had irregular intestinal ulcers in the rectum, sigmoid colon and ileocecal junction and longitudinal ulcers in the transverse colon and descending colon. Nodules and erythema were present in the sigmoid colon, descending colon and rectum of patient 2. Patient 3 had an intestinal ulcer in the colon and cobblestone-like polyposis. A patched mucosal ulcer was observed from the rectum to the ascending colon in patient 4. Patients 5, 6 and 7 had colon and rectal ulcers, inflammatory polyps and rectal stenosis (Fig. 2a–d).

**Fig. 3:**
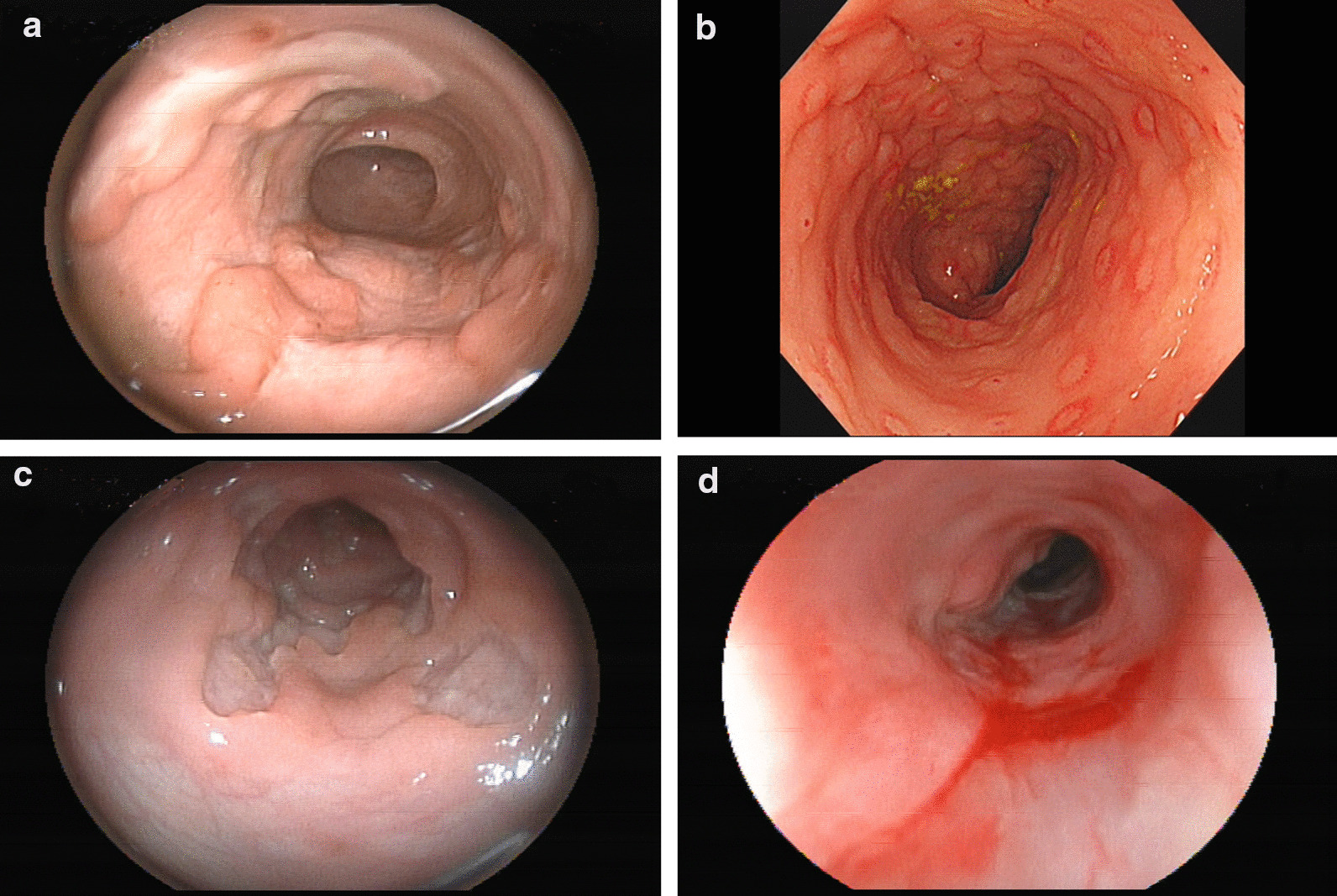
Colonoscopy images of VEOIBD patients with *IL10RA* gene mutations. **a** Longitudinal ulcer of the transverse colon in case 1. **b** Presence of nodules and erythema of the sigmoid colon in case 2. **c** Irregular ulcer of the descending colon in case 3. **d** Rectal stenosis and multiple polyps in case 5

The typical imaging findings of these patients were evaluated. Radiological examination showed colon and rectal disease in enhanced CT and MRI examinations (Fig. 3a–c).

### Treatment and follow-up

Six of the patients agreed to thalidomide treatment. Two patients agreed to mesalazine treatment. Two patients agreed to infliximab infusion and stopped because of several allergies. Two patients underwent HSCT. Patient 7 underwent enterostomy because of severe infection and perianal lesions. Patients 1 and 3 also had cytomegalovirus (CMV)-related inflammation and recovered after antiviral treatment.

After follow-up, patients 5 and 6 were in remission after HSCT, and Sanger sequencing confirmed *IL10RA* gene repair. Patient 2 died of sepsis after HSCT (Fig. 3D). Patients 2, 4 and 7 were waiting for HSCT, while patient 1 had no intention of undergoing HSCT because of the stability of the disease.

### Literature review

We searched the literature from January 2009 to June 2020 in the PubMed and HGMD databases, and only one paper on large deletions in the *IL10RA* gene was reported by Engelhardt et al. [13]. An Arabic patient was found to have deletions in exons 1, 2, and 3 of *IL10RA* (Ex1_3del; homozygous). The onset age for this child was 2 months. No transplantation was performed until 1 year of age at the time of manuscript drafting. The main clinical findings were severe colitis and severe otitis media/urinary tract infections. This infant received therapy with prednisone and azathioprine and showed *Candida* species-induced septicemia. Follow-up showed that the infant had ongoing colitis with no remission. To date, no additional article has reported large deletions of the *IL10RA* genes, particularly in the East Asian population, where the point mutations c.301C > T (p. R101RW) and c.537 G > A (p. T179T) of *IL10RA* are common.

## Discussion

VEOIBD patients with *IL10/IL10R* signaling pathway deficiency are characterized by early onset refractory diarrhea and severe infectious diseases, oral ulcers, and perianal diseases (abscess, fistula formation, fissure, and skin tags) early in life with severe evolution in some cases [14, 15].

Glocker et al. first reported patients with *IL10R* signaling defects [16]. Since then, other defects in *IL10* and its receptors *IL10RA* and *IL10RB* have been reported [13, 15, 17–21]. Our group also reported the phenotype and genotype of *IL10R*-mutated VEOIBD patients in our collaborative study. We identified c.301C > T (p. R101RW) and c.537 G > A (p. T179T) as common mutations of *IL10RA* in the East Asian population [9]*.*

WES and targeted gene panel sequencing have emerged as powerful tools to screen genes of interest [22, 23]. Point mutations, small fragment deletions and insertions can be identified by WES and targeted gene panel sequencing. Although point mutations and small region deletions are easily detected with WES, large fragment deletions may be missed. The failure to identify associations between IL10 variants and more common forms of Crohn’s disease may be explained by the current limitations of NGS bioinformatics analysis. Small region deletions and point mutations such as deletions and insertions can be found by Sanger sequencing; unfortunately, large fragment deletions or copy number variations are easily missed [24]. Large fragment deletions of genes cause the loss of protein function. With the development of bioinformatics analysis, not only point mutations but also large fragment deletions can be identified. Large fragment deletions of genes can be further confirmed by multiplex ligation-dependent probe amplification or QT-PCR.

For large fragment deletions of the *IL10RA* gene, Engelhardt et al. first reported an exon 1_3 large deletion in an Arabic patient. In the current study, we found a novel large deletion of exon 1 of IL10RA in 7 patients for the first time. This 333-bp deletion of exon 1:117857034–11857366 was the main mutation form in these patients. We further confirmed a mutation at a possible hotspot of the *IL10RA* gene large fragment deletions. Regarding VEOIBD patients, particularly those with perianal diseases, *IL10/IL10R*-related monogenic diseases are often suspected [25]. In addition to point mutations, small fragment deletions and insertions and large fragment deletions should be considered. Other methods, such as multiplex ligation-dependent probe amplification or QT-PCR, should also be used [20, 26]. Identification of the large fragment deletions of the IL10RA gene has an important effect on clinical practice. For those patients, identifying large fragment deletions of the IL10RA gene helps the genetic diagnosis of VEOIBD. Furthermore, after confirming the compound mutation of IL10RA with a point mutation and large fragment deletions, these children can undergo HSCT to cure this disease. In our study, among the 7 patients with large fragment deletions, 3 accepted HSCT, 2 were in remission, and 3 were awaiting HSCT. Furthermore, the confirmation of a compound mutation of IL10RA with a point mutation and large fragment deletions helps the prenatal diagnosis and consultation for parents and perinatal geneticists.

Children with VEOIBD and *IL10/IL0R* signaling pathway deficiency have more extensive, severe, and refractory disease than older children and adults with IBD. The treatment for these patients is challenging [27]. In this study, large fragment deletions of the *IL10RA* gene caused severe intestinal complications. To determine the differences and similarities in these patients, we compared patients with large deletions in exon 1 with the control group. There was no significant difference except in the time of diarrhea and the percentage of eczema. These two kinds of mutations all cause deleterious effects. The patients with large fragment deletions of the *IL10RA* gene received mesalazine, thalidomide, infliximab infusion, HSCT, and enterostomy. Thalidomide and enterostomy have shown to be efficient treatments for remission in previous studies. HSCT has been proven to be a radical cure. Furthermore, two patients had CMV infection because of the high expression of CMV DNA. Therefore, for *IL10RA*-mutated VEOIBD, opportunistic infection should be considered because of immunodeficiency disorders. Our study has several limitations. First, comprehensive functional studies are lacking concerning these patients. Additionally, the comparison with other mutations of IL10RA may have a bias because of the small number of VEOIBD patients with large fragment deletions in the *IL10RA* gene.

## Conclusions

Our study is the first to report large fragment deletions in the *IL10RA* gene in Chinese VEOIBD patients. This study expands the spectrum of Chinese VEOIBD patients with *IL0RA* gene mutations.

## Data Availability

The datasets generated and/or analyzed during the current study are available in the ClinVar repository [Accession Nos. SCV001547199, SCV001519656].

## Abbreviations

ACMG: American College of Medical Genetics and Genomics
BMI: Body mass index
HFA: Height for age
HSCT: Hematopoietic stem cell transplantation
IL10: Interleukin 10
IQR: Interquartile range
SD: Standard deviation
NGS: Next-generation sequencing
PCR: Polymerase chain reaction
VEOIBD: Very early onset inflammatory bowel disease
WFA: Weight for age
WHO: World Health Organization
